# IDENTIFYING COMMUNITY PARTICIPATION CHALLENGES FOR ADULTS AGING WITH MOBILITY AND VISION DISABILITIES

**DOI:** 10.1093/geroni/igz038.2790

**Published:** 2019-11-08

**Authors:** Maurita T Harris, Lyndsie M Koon, Elena T Remillard, Wendy A Rogers

**Affiliations:** 1 University of Illinois at Urbana Champaign, Champaign, Illinois, United States; 2 Georgia Institute of Technology, Atlanta, Georgia, United States

## Abstract

There are growing numbers of older adults with mobility and vision disabilities acquired in early to mid-life who are a part of a population described as “aging with disability”. For these individuals, the addition of normative age-related declines (e.g., vision loss, arthritis) on top of a long-term disability can create extensive barriers to community participation. We present findings on activity challenges with community participation among older adults with long-term vision and mobility disabilities (N=120) from the Aging Concerns, Challenges, and Everyday Solution Strategies (ACCESS) interview study. Results provide detailed insights on the specific task-based challenges experienced when engaging in one’s community (e.g., going to entertainment events, doing activities with a group or organization, and participating in religious services and activities) as well as the solutions and strategies employed to overcome those challenges. Findings provide guidance for the design of supportive technologies that promote participation and independence for this understudied population.

